# DC-FusionGNN: A Dual-Channel Framework Integrating Global Self-Attention and Local Topology Learning for Identifying Key Resistance Genes Against *Fusarium graminearum* Infection in Maize

**DOI:** 10.3390/plants15101540

**Published:** 2026-05-18

**Authors:** Yinfei Dai, Mengjiao Qiao, Jie Fan, Shihao Lu, Enshuang Zhao, Yuheng Zhu, Hanbo Liu, Hao Zhang

**Affiliations:** 1 College of Computer Science and Technology, Jilin University, Changchun 130012, China; daiyf23@mails.jlu.edu.cn (Y.D.); zhaoes22@mails.jlu.edu.cn (E.Z.); yhzhu23@mails.jlu.edu.cn (Y.Z.); hbliu24@mails.jiu.edu.cn (H.L.); 2 College of Computer Science and Technology, Changchun University, Changchun 130022, China; qiaomengjiaolemon@outlook.com (M.Q.); woxinwuxin@163.com (J.F.); 231501510@mails.ccu.edu.cn (S.L.)

**Keywords:** graph neural networks, dual-channel feature fusion, gene regulatory network, maize, *Fusarium graminearum*

## Abstract

*Fusarium graminearum* infection of maize induces complex transcriptional reprogramming, yet existing differential-expression and local graph convolutional approaches struggle to capture long-range and multi-scale regulatory dependencies. We propose DC-FusionGNN, a dual-channel fusion graph neural network for key resistance-gene identification. Based on the transcriptome dataset GSE174508, we first construct a comprehensive gene interaction network by integrating a WGCNA co-expression network with a STRING-based interaction network. The left channel combines structure-aware propagation with a Transformer-based global self-attention mechanism to model long-range cross-module dependencies, while the right channel couples GraphSAGE with a GCN to capture local topology and neighborhood heterogeneity. Embeddings from the two channels are concatenated to form a unified gene representation, trained via self-supervised link prediction. Compared with baseline graph neural networks, DC-FusionGNN achieves competitive and overall improved performance across multiple metrics, and robustness and independent cross-species (rice, GSE39635) experiments further confirm its stability and generalization ability. GO and KEGG enrichment analyses show that the top-ranked candidate genes are significantly enriched in plant defense responses, hormone signaling, and secondary metabolism, supporting the biological relevance of the model’s predictions.

## 1. Introduction

*Maize (Zea mays)* is one of the most important food crops in the world, and its yield and quality are directly related to food security and sustainable agricultural development [1]. Gibberella stalk rot (GSR) is a widespread and serious fungal disease in maize production, mainly caused by pathogens [2] such as *Fusarium graminearum*. This disease usually occurs in the middle and late stages of maize growth, which can lead to stalk tissue rot, plant lodging and poor grain filling, and in severe cases, significant yield reduction. In addition, *Fusarium graminearum* can also produce a variety of fungal toxins, which pose a potential threat to food security and human and animal health [3]. Therefore, in-depth analysis of the molecular response mechanism of maize in the process of *Fusarium graminearum* infection is of great theoretical significance and practical value for the prevention and control of Gibberella stalk rot.

Maize resistance to Gibberella stalk rot is a typical quantitative trait, which is regulated by multiple genes and involves the joint participation of multiple biological pathways [4] such as plant defense response, signal transduction and secondary metabolism. In recent years, with the rapid development of high-throughput sequencing technology, transcriptome sequencing (RNA-seq) has been widely used to analyze the dynamic transcriptional response characteristics of maize during *Fusarium graminearum* infection [5]. Related studies have identified a large number of differentially expressed genes (DEGs) closely related to disease resistance, including candidate genes encoding NLR-type disease resistance proteins, WRKY transcription factors, and key enzymes in the phenylpropane metabolic pathway. These studies [6,7,8] have significantly deepened the understanding of the *maize-Fusarium graminearum* interaction mechanism at the molecular level and provided important clues for the discovery of disease resistance-related functional genes. However, existing studies are mostly limited to single-gene or single-pathway levels [9,10], mainly relying on differential expression analysis, and lack a holistic reconstruction of the infection response gene network from a systems biology perspective and precise identification of key regulatory nodes. Network biology methods provide a powerful framework for analyzing complex biological processes at the systems level. Weighted Gene Co-expression Network Analysis (WGCNA) identifies gene modules significantly associated with specific biological states (such as pathogen infection) by mining co-expression patterns of genes in different samples, and further screens potential core regulatory factors [11,12]. Meanwhile, the STRING database integrates multi-source evidence from experimental validation, literature mining, and cross-species homology inference, providing high-confidence molecular functional association information. Although the original STRING records are protein-based, they can be reliably converted into gene-based functional association networks through the Ensembl gene-protein [13] official mapping relationship. Integrating condition-specific co-expression networks with prior functional association networks not only preserves the dynamic response characteristics under *Fusarium graminearum* infection but also introduces evolutionarily conserved biological knowledge [14], thus more comprehensively and robustly reconstructing the gene regulatory network topology in maize disease resistance.

Traditional network analysis methods [15] typically rely on topological indicators such as degree centrality and betweenness centrality to assess the importance of genes in the network [16]. However, these methods are mainly based on static network structures, making it difficult to effectively integrate the dynamic expression characteristics of genes under specific biological conditions (such as disease states, developmental stages, or environmental stimuli), thus limiting their accuracy and biological relevance in identifying key functional genes. In recent years, Graph Neural Networks (GNNs), as a deep learning method specifically designed for graph-structured data, have shown significant advantages in the analysis of complex biological networks. GNNs, by iteratively propagating and aggregating information from neighboring nodes in the graph, can learn the low-dimensional embedding representation of each gene, preserving not only the topological characteristics of the network but also integrating node attribute information [17] (such as gene expression levels), thereby effectively capturing potential nonlinear interactions between genes [18]. This capability provides a novel approach and technical path for accurately identifying key regulatory genes in specific biological contexts.

Although previous studies [19,20] have attempted to elucidate the response mechanism of maize to *Fusarium graminearum* infection at the transcriptome level, research on *Fusarium graminearum* stalk rot, which systematically integrates differentially expressed gene co-expression networks with protein interaction prior information from the STRING database [21] and introduces graph neural network models at the gene level to identify key disease resistance-related genes, remains limited. In particular, there is a lack of systematic exploration in deep learning modeling at the gene network level, combining node expression features.

In this study, we screened differentially expressed genes related to *Fusarium graminearum* stalk rot based on transcriptome data of maize infected with *Fusarium graminearum* from the GEO database and constructed a gene co-expression network using WGCNA. Simultaneously, we constructed a maize gene interaction network by combining high-confidence protein interaction information from the STRING database. Based on this, we used standardized gene expression levels as node features and the integrated gene network as graph structure input to construct a graph neural network model, and then trained the gene network through a link prediction task.

Finally, genes were ranked based on a representation-derived score obtained from the model output, where the L2 norm of the node embedding was used as a quantitative measure of its prominence in the learned feature space. It should be noted that this score reflects a mathematical property of the learned representation rather than a direct measure of biological regulatory importance. Functional enrichment analysis was then performed on the top-ranked candidate genes to identify those potentially involved in resistance to maize scab during *Fusarium graminearum* infection.

## 2. Materials and Methods

**Figure 1:**
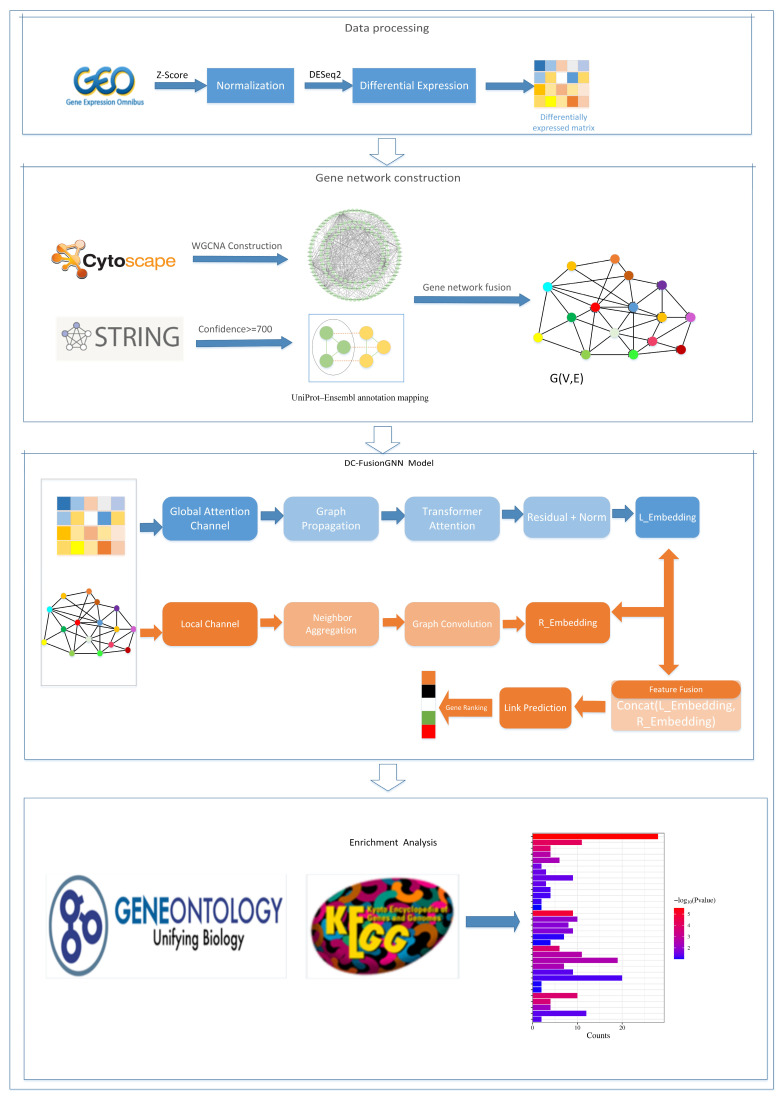
Overall technical workflow. The pipeline comprises four stages: (1) Data Processing: Differential expression analysis of GEO transcriptomic data using DESeq2 based on raw count data, followed by log-transformation and Z-score normalization of the selected differentially expressed genes for downstream analysis; (2) Network Construction: Fusion of WGCNA co-expression and STRING PPI networks (confidence ≥ 0.7) to generate graph *G* (*V*,*E*); (3) Model Prediction: Feature extraction via the dual-channel DC-FusionGNN for link prediction and candidate gene prioritization; (4) Functional Analysis: GO and KEGG enrichment analysis of prioritized candidate genes.

To systematically investigate the gene regulation mechanisms in maize during *Fusarium graminearum* infection, this paper constructs a complete analytical framework, including data preprocessing, gene co-expression network construction, graph neural network modeling, and functional enrichment analysis. The overall process is shown in Figure 1.

Specifically, firstly, transcriptome data were standardized and differentially expressed genes were screened. Then, a gene co-expression network was constructed using the WGCNA method and fused with the STRING database (v12.0) to form a overall gene network. Based on this, a dual-channel fusion graph neural network model was constructed for feature learning and key gene identification. Finally, functional enrichment analysis was performed on the selected candidate genes to reveal their potential biological functions.

### 2.1. Data Collection and Processing

#### 2.1.1. Data Processing

The transcriptome data used in this study came from the publicly published dataset GSE174508 in the NCBI Gene Expression Omnibus (GEO) database. This dataset used the *maize (Zea mays)* inbred line B73 as the research material, and maize stem tissue was used to systematically record the transcriptome response changes of maize during *Fusarium graminearum* infection. Three infection time points were set up: 0 h, 12 h, and 24 h after infection. Each time point contained three biological replicates. The dataset initially contained a total of 9 samples with dynamic gene expression characteristics from the early to mid-stages of pathogen infection.

To ensure the reliability of differential expression analysis and the stability of subsequent network analysis and graph neural network modeling, the original raw gene expression count matrix was first used for differential expression analysis using DESeq2 [22]. After identifying differentially expressed genes, preprocessing steps were applied for downstream analysis, including filtering out genes with an expression value of 0 in all samples and retaining only those expressed in at least one sample. Subsequently, the expression data were transformed using log_2_(x + 1). For network construction based on WGCNA, the log-transformed expression data were used directly to preserve the intrinsic gene–gene correlation structure, and the analysis was restricted to differentially expressed genes to reduce noise and computational complexity. In contrast, Z-score standardization across samples was applied only for graph neural network modeling, where gene expression values were centered and scaled to improve numerical stability and feature comparability. The processed data were then used for their respective downstream analyses.

To reduce noise and focus on key response genes of maize to *Fusarium graminearum* infection, this study performed differential expression analysis on transcriptome data [23]. Based on the standardized expression matrix, the gene expression levels at different infection time points were compared. The samples before infection (0 h) were used as the control group, and differential analysis was performed with the samples after infection (12 h and 24 h). Statistical tests were used to evaluate gene expression changes under different conditions [24], and the results of multiple tests were corrected. The screening criteria for significantly differentially expressed genes were set as follows: corrected *p* < 0.05, |log_2_FoldChange| ≥ 1. The differentially expressed genes (DEGs) finally selected were used for subsequent WGCNA co-expression network construction and graph neural network model analysis.

#### 2.1.2. Construction of Weighted Gene Co-Expression Network

Based on the standardized expression matrix of the screened differentially expressed genes, a gene co-expression network of maize under *Fusarium graminearum* infection was constructed using Weighted Gene Co-expression Network Analysis (WGCNA) [25]. First, the gene expression matrix was transposed so that samples served as rows and genes as columns, satisfying the input data structure requirements of the WGCNA method. Subsequently, the goodSamplesGenes function within the WGCNA package was employed for quality control to automatically detect and remove potentially outlier samples or genes [26], ensuring that the data used for network construction possessed high integrity and consistency.

Based on the expression data that passed quality control, a soft-thresholding power of 12 was selected to construct the weighted gene co-expression network. The optimal soft-threshold was determined using the scale-free topology criterion implemented in WGCNA, where the scale-free topology fit index (R^2^) and mean connectivity were evaluated across a range of candidate powers. A power of 12 was chosen as it achieved a satisfactory scale-free topology fit while maintaining sufficient network connectivity. This soft-threshold was then used to transform the Pearson correlation matrix [27] between genes into a weighted adjacency matrix. The power transformation assigns higher connection weights [28] to strongly correlated gene pairs while attenuating weak correlations, thereby enhancing the robustness of the co-expression network.

After obtaining the adjacency matrix, the Topological Overlap Matrix (TOM) [29] was calculated to comprehensively consider both the direct interactions between gene pairs and the extent of overlap in their shared neighbors. The TOM effectively reduces noise introduced by spurious correlations, thereby providing a more robust representation of gene co-expression relationships within the network.

To facilitate the subsequent construction of the Graph Neural Network model, the TOM was further filtered using a thresholding strategy. Specifically, only gene pairs with a topological overlap value ≥ 0.1 were retained as network edges [30]. This threshold was selected in accordance with commonly adopted practices in WGCNA-based analyses, aiming to balance network sparsity and biological relevance. Lower thresholds tend to introduce weak and potentially spurious associations, whereas higher thresholds may lead to overly sparse networks and the loss of biologically meaningful interactions. Therefore, the chosen cutoff provides a reasonable trade-off between preserving essential network structure and reducing noise.

**Figure 2:**
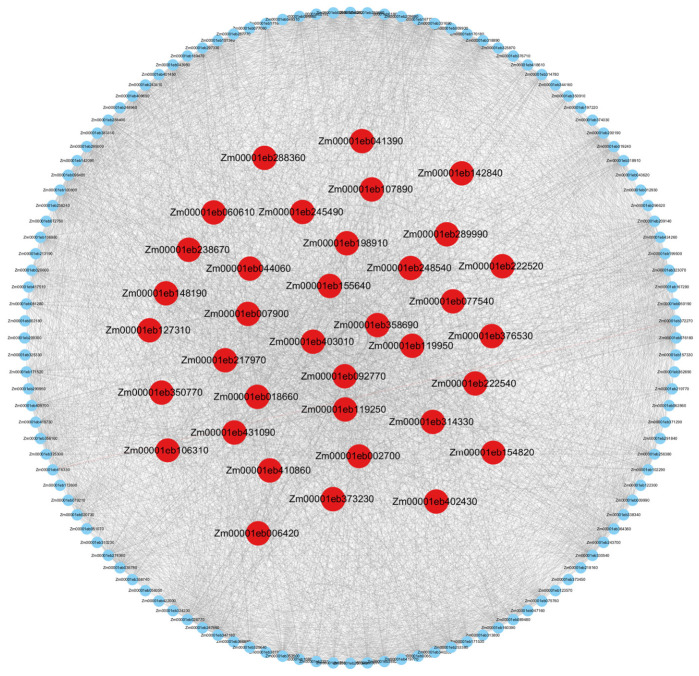
Weighted gene co-expression network constructed based on WGCNA. This figure shows a representative overview of the overall co-expression network topology. Red nodes represent hub genes selected by WGCNA.

We constructed a weighted gene co-expression network through the above steps [31]. In order to visualize the above network structure and intuitively show the co-expression relationship between genes, we constructed a partial network diagram, as shown in Figure 2.

Finally, the screened gene pairs and their corresponding TOM weight information were exported as the edge file of the co-expression network. Simultaneously, all genes involved in the network construction were aggregated to generate the corresponding node file, providing the data foundation for using the co-expression network as a prior structure input for the Graph Neural Network model.

#### 2.1.3. STRING Protein-Protein Interaction Network and Gene-Level Mapping

The protein-protein interaction network data were obtained from the STRING database, and the selected species was *maize* (*Zea mays*, Taxonomy ID: 4577). First, the protein-protein interaction files [32] corresponding to this species were downloaded to obtain the original protein-protein interaction relationships represented by STRING protein IDs and their combined confidence scores.

**Figure 3:**
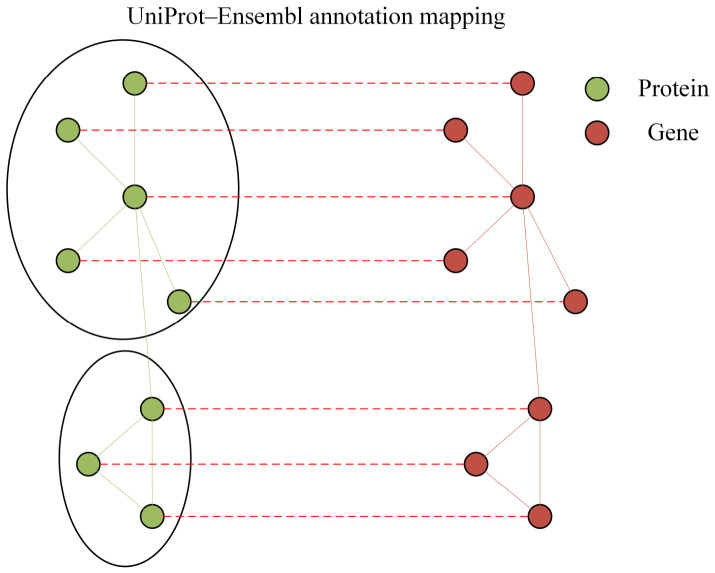
Schematic diagram of the UniProt–Ensembl annotation mapping process. This figure illustrates the conversion of protein-protein interaction data from the STRING database into a maize gene (Zm ID) network. The green nodes on the left represent STRING protein IDs, while the red nodes on the right correspond to maize gene IDs. By establishing a one-to-one correspondence between UniProt TrEMBL IDs and Ensembl Gene IDs [36], interaction information at the protein level is accurately mapped back to the gene level, retaining only successfully mapped nodes for subsequent analysis.

Since the interaction information in the STRING database is based on protein-level representations, whereas the transcriptomic analysis and subsequent network modeling in this study are gene-centric, a UniProt–Ensembl annotation mapping table was introduced to convert STRING protein identifiers to maize gene identifiers (Zm IDs). During the mapping process, the species prefix (“4577.”) was first removed from STRING protein IDs. The mapping between UniProt TrEMBL protein IDs and Ensembl Gene IDs was then established based on available annotation resources. It should be noted that protein-to-gene relationships are not strictly one-to-one due to the presence of isoforms and annotation redundancy [33]; therefore, multiple proteins may map to the same gene, or a single protein may correspond to multiple gene entries. To ensure consistency and avoid ambiguity in network construction, we retained only successfully mapped protein pairs and consolidated redundant mappings at the gene level by merging interactions corresponding to the same gene pairs. This procedure ensures a coherent gene-level interaction network while minimizing potential biases introduced by mapping multiplicity. The overall mapping workflow is illustrated in Figure 3.

To improve the reliability and biological credibility of the network [34], high-confidence interaction relationships with a STRING comprehensive confidence score of not less than 700 were further screened. Subsequently, the selected protein interaction pairs were subjected to intersection screening with the gene set in the transcriptome expression matrix, retaining only gene pairs that actually appeared in the expression data to ensure the consistency between the protein interaction network and the expression data at the gene level [35].

Finally, a high-confidence maize gene interaction network represented by Zm ID was obtained and saved as a side file for subsequent integration with co-expression networks and as input to graph neural network models.

#### 2.1.4. Gene Network Fusion

To comprehensively characterize the gene regulation and functional relationships in maize during *Fusarium graminearum* infection, this study integrated a co-expression network and a protein interaction network [37] at the gene level to construct a comprehensive gene network as the structural basis for subsequent graph neural network analysis.

First, the edge files of the gene co-expression network constructed based on WGCNA and the edge files of the protein interaction mapping gene network [38] constructed based on the STRING database were obtained. Since both networks are undirected, all gene pairs were standardized before network fusion, that is, the two endpoints of each undirected edge were rearranged in alphabetical order to ensure that the same gene pair is represented in a unique form, thereby avoiding duplicate edges caused by different node orders.

**Figure 4:**
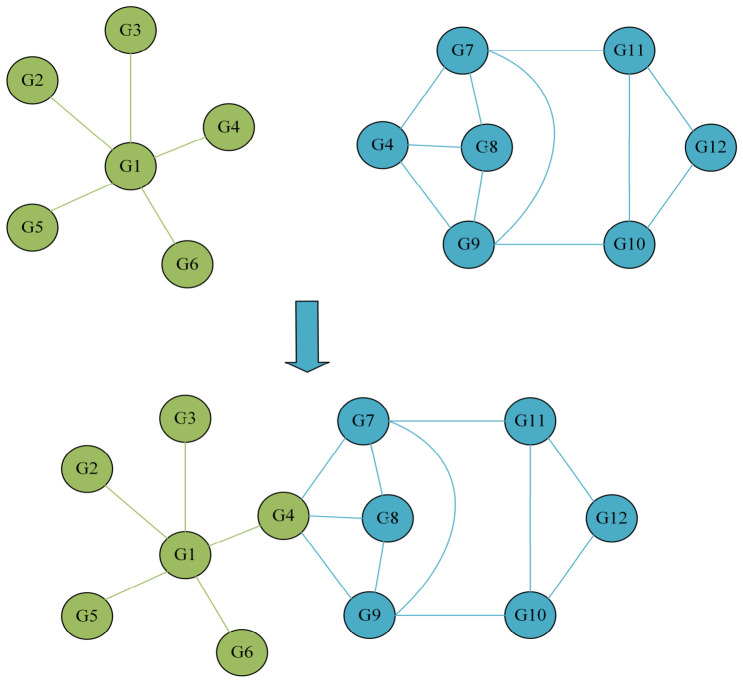
Illustration of the gene network integration process. The WGCNA-based co-expression network and the STRING-based protein interaction network were merged by unifying gene pairs and removing duplicate edges. The resulting network retains both co-expression relationships and protein–protein interactions, providing a comprehensive representation of gene associations.

After standardization, the edges in the WGCNA and STRING networks were deduplicated, and the two sets of edges were merged. During the merging process, all gene associations from the co-expression network and the protein interaction network were retained, while duplicate gene pairs were removed, as shown in Figure 4. Finally, a comprehensive gene network without redundant edges was obtained. This fused network retains the co-expression relationships inferred from transcriptome data and introduces high-confidence protein interaction information [39,40] from a priori knowledge base, thus achieving complementarity between data-driven and knowledge-driven approaches.

The final integrated gene network is constructed with Zm IDs as nodes and gene pairs as edges, and is saved in a standardized edge file format for use as structural input to subsequent graph neural network models.

### 2.2. Model Framework

**Figure 5:**
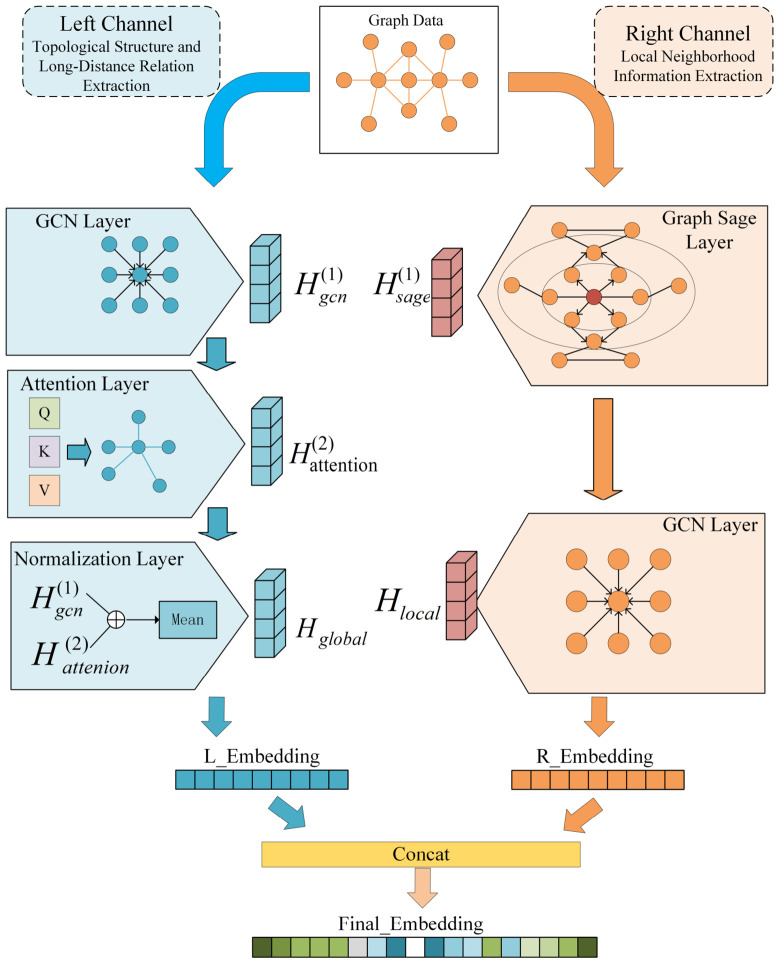
Dual-channel fusion graph neural network for gene representation learning. This framework includes a channel based on global attention and a channel based on local topology. The global channel uses a graph attention mechanism to extract topological information and model global dependencies, while the local channel captures neighborhood structural features through information propagation and aggregation. The generated embeddings (L_Embedding and R_Embedding) are concatenated to form a unified embedding representation (final embedding).

This study proposes a dual-channel graph neural network model based on the fusion of multi-source gene networks to identify key regulatory genes in maize during *Fusarium graminearum* infection from a complex gene interaction system. The overall framework of the model is shown in Figure 5. Its core idea is to learn gene representations through a graph neural network by fusing expression-driven networks and prior interaction networks, thereby characterizing the potential importance of genes in the network regulatory structure.

In the model input layer, genes are modeled as nodes in the graph, with node features represented by standardized gene expression feature vectors. To construct a reasonable graph structure, the model introduces two complementary types of gene network information: one is a weighted co-expression network (WGCNA) based on differentially expressed genes, used to characterize the expression synergies closely related to the disease response under *Fusarium graminearum* infection conditions; the other is a network of STRING protein interaction mapping genes constructed based on the complete set of standardized expressed genes, used to incorporate existing biological interaction prior information.

Based on this, the model constructs a comprehensive gene network by uniformly aligning the node space and fusing the edges of the two networks, and uses this as the topological input of the graph neural network. This integrated network simultaneously incorporates conditionally relevant expression-driven information and stable prior interaction structures, providing more comprehensive and robust structural constraints for model learning.

In the representation learning phase, the model uses a graph neural network to propagate and aggregate features from the integrated gene network. By iteratively integrating the expression features of neighboring genes with network topology information, it learns the low-dimensional embedding representation of each gene in the global regulatory network. This process effectively captures high-order dependencies between genes, overcoming the limitation of traditional differential expression analysis that only focuses on single-gene changes.

Finally, based on the learned gene embedding representations, the model quantitatively evaluates the regulatory potential of genes in the integrated network and thereby screens candidate genes that may play key regulatory roles in *Fusarium graminearum* infection.

### 2.3. Model Design of the Dual-Channel Fusion Graph Neural Network

Addressing the characteristics of the maize gene regulatory network during *Fusarium graminearum* infection—namely, its complex structure, diverse regulatory hierarchies, and highly nonlinear expression dependencies—this paper proposes a dual-channel fusion graph neural network model based on a comprehensive gene network. By constructing two feature propagation channels in parallel, each with distinct modeling preferences, the model performs representation learning on genes from two complementary perspectives: global expression dependency modeling and local topological structure aggregation. This approach enables a more comprehensive characterization of the potential regulatory roles of genes within the disease response network.

As shown in Figure 5, the model adopts the design concept of “multi-source network construction → dual-channel feature learning → feature fusion → self-supervised training and gene scoring”. Its core innovation lies in the fact that by using dual-channel parallel modeling and feature-level fusion mechanism, it effectively integrates expression-driven information and network structure priors, breaking through the limitation of a single graph neural network in modeling complex biological regulatory systems.

#### 2.3.1. Model Input Representation and Formal Definition of Problem

At the model input stage, this study formalizes the integrated gene network as an undirected graph:

(1) G=(V,E)

where $V=\left\{v_{1},v_{2},\ldots ,v_{N}\right\}$, represents the set of gene nodes. The node feature matrix is denoted as:

(2) $$X=[x_{1},x_{2},\ldots ,x_{N}{]}^{⊤}\in R^{N\times d}$$

where $x_{i}\in R^{d}$ represents the standardized expression feature vector of the *i*-th gene across different infection time points.

The learning objective of the model is to construct a mapping function via the graph neural network:

(3) $$f_{\theta }:\left(G,X\right)\to Z$$

where denotes the learned low-dimensional gene embedding representations, used to characterize the potential functional states and structural importance of genes within the integrated regulatory network.

#### 2.3.2. Graph Feature Learning Path Based on Global Attention and Structure-Aware Propagation

The design goal of this module is to improve the modeling paradigm of traditional graph convolutional models (which rely solely on local neighborhood aggregation) by explicitly characterizing cross-module and long-range expression dependencies between genes. To this end, based on graph structure modeling, the left channel of the model integrates a Transformer-based global attention mechanism and a structure-aware feature propagation strategy. This constructs a gene representation learning channel that combines global modeling capabilities with graph structure constraints.

In terms of implementation, this channel consists of two sequential components: first, a structure-aware graph feature pre-propagation module is employed to smooth and enhance node features; subsequently, a global feature interaction module based on a self-attention mechanism is utilized to model gene expression relationships.

- Structure-Aware Graph Feature Pre-Propagation

Let the fused gene network be represented as an undirected graph G=(V,E), where V denotes the set of gene nodes and E represents the regulatory or interaction relationships between genes. Given the initial node feature matrix:

(4) $$X\in R^{N\times d}$$

The left channel first introduces a structure-aware graph feature propagation module to perform preliminary aggregation of node features.

This module completes one round of graph convolution-style propagation based on the normalized adjacency matrix, which can be formulated as:

(5) $$\tilde{X}=D^{-\frac{1}{2}}AD^{-\frac{1}{2}}X$$

where A is the adjacency matrix of the graph and D is the corresponding degree matrix. This operation is implemented using the PyTorch (v2.1.0) Geometric framework, where sparse graph structures are efficiently represented using SparseTensor and matrix multiplication (matmul) operations to perform weighted aggregation of expression features from neighboring genes. Subsequently, the propagated features are linearly mapped together with the initial features:

(6) $$\;H^{\left(0\right)}=\sigma \left(W_{0}\tilde{X}\right)$$

where $W_{0}$ represents learnable parameters and σ(⋅)  denotes the ReLU activation function. This step corresponds to the linear transformation and activation operations on input features in the code, providing stable feature representations for subsequent global attention modeling.

- Global Feature Interaction Based on Self-Attention Mechanism

After obtaining the initial hidden representation $H^{\left(0\right)}$, the left channel further incorporates a Transformer-based self-attention mechanism to explicitly model expression dependencies among all gene nodes. Unlike traditional Graph Attention Networks that compute attention only within local neighborhoods, this module executes attention calculations across the entire node space, thereby capturing potential long-range regulatory relationships.

In the *l*-th layer, node features are first mapped into Query, Key, and Value vectors, respectively:

(7) $$Q^{\left(l\right)}=H^{\left(l\right)}W_{Q},K^{\left(l\right)}=H^{\left(l\right)}W_{K},V^{\left(l\right)}=H^{\left(l\right)}W_{V\;}$$

where $W_{Q},W_{K},W_{V}$ are learnable linear mapping matrices. Subsequently, the attention weight between node i and node j  is defined as:

(8) $$\;\alpha_{ij}^{\left(l\right)}=\frac{\exp\left(\frac{(Q_{i}^{\left(l\right)}{)}^{⊤}K_{j}^{\left(l\right)}}{\sqrt{d}}\right)}{\sum_{k=1}^{N}\;\exp\left(\frac{(Q_{i}^{\left(l\right)}{)}^{⊤}K_{k}^{\left(l\right)}}{\sqrt{d}}\right)}$$

Based on the aforementioned attention weights, node representations are updated through global weighted aggregation:

(9) $$\;{\hat{H}}_{i}^{\left(l+1\right)}=\sum_{j=1}^{N}\;\alpha_{ij}^{\left(l\right)}V_{j}^{\left(l\right)}$$

This process enables each gene node to comprehensively consider the expression states of all other genes when updating its representation, thereby effectively modeling system-level synergistic response features.

- Residual Connections and Normalization Strategies

To mitigate the vanishing gradient problem during the training of deep models and to preserve original feature information, the left channel introduces residual connections after each layer of attention update:

(10) $$\;H^{\left(l+1\right)}=\frac{{\tilde{H}}^{\left(l+1\right)}+H^{\left(l\right)}}{2}$$

Simultaneously, the model further employs Layer Normalization to standardize node representations, yielding the final embedding:

(11) $$z^{global}=LayerNorm\left(H^{\left(l+1\right)}\right)$$

This enhances training stability and improves the model’s generalization capability. This design is strictly consistent with the LayerNorm and residual averaging operations in the code.

Through the above structure, the left channel achieves global expression dependency modeling under structural constraints on the integrated gene network. On one hand, the initial graph feature propagation step introduces structural information from the gene interaction network; on the other hand, the global self-attention mechanism enables the model to capture potential synergistic regulatory patterns between different functional modules.

This design is particularly suitable for the widespread phenomenon of systemic transcriptional reprogramming during *Fusarium graminearum* infection, making the left channel more inclined to learn globally regulated key genes, thereby forming a complement to the local structure modeling of the right channel.

#### 2.3.3. Local Topological Structure Feature Learning Based on GraphSAGE and GCN

To systematically characterize the local topological structure features of gene nodes and their neighborhood regulatory patterns within the integrated gene network, this study constructs a local structure feature learning module in the right channel of the dual-channel model. This module combines GraphSAGE with Graph Convolutional Networks (GCN). Taking the comprehensive gene network as input, this channel models the structural positions and functional associations of genes within local interaction networks through explicit neighborhood feature aggregation and graph-structure-based normalized message propagation. This approach addresses the insufficiency of the left channel in characterizing local structures due to its focus on global dependency modeling.

The right channel shares the same input as the left channel, namely the comprehensive gene network:

G=(V,E)

where V represents the set of gene nodes with ∣V∣=N, and E denotes the set of interaction edges between genes. For any gene node $v_{i}\in V$, its initial node feature vector is represented as:

(12) $$x_{i}\in R^{F}$$

where F is the dimension of the node features, corresponding to the standardized expression features of the gene across different samples or conditions.

In the first stage of the right channel, GraphSAGE is introduced to aggregate features from the node and its local neighborhood. For a node $v_{i}$, let its first-order neighborhood node set be defined as N(i). GraphSAGE first summarizes the features of neighborhood nodes via an aggregation function to obtain the neighborhood representation for node $v_{i}$:

(13) $$h_{N\left(i\right)}=AGGREGATE\left(\left\{x_{j}|j\in N\left(i\right)\right\}\right)$$

where AGGREGATE(⋅) denotes the neighborhood aggregation function. In this study, this function adopts the form of mean aggregation [41], calculating the element-wise average of the neighborhood node feature vectors to obtain stable local expression statistics.

Subsequently, the node’s own feature and its neighborhood aggregated feature are concatenated along the feature dimension. A linear mapping followed by a nonlinear activation function is then applied to generate the intermediate representation of the node:

(14) $$h_{i}^{\left(1\right)}=\sigma \left(W_{s}\cdot \left[\begin{array}{c} x_{i}∥h_{N\left(i\right)} \end{array}\right]\right)$$

where the symbol ∥ denotes the vector concatenation operation; $W_{s}\in R^{F^{\prime }\times 2F}$ is a learnable weight matrix used for the linear transformation of the concatenated features; “⋅” represents matrix multiplication; and σ(⋅) is a nonlinear activation function, implemented here as the ReLU function to enhance the model’s nonlinear expressive power. Through this process, while retaining its own expression features, the node representation incorporates synergistic expression information from local neighborhood genes, thereby effectively characterizing the functional heterogeneity of genes within local regulatory modules.

After completing the neighborhood feature aggregation based on GraphSAGE, the right channel further introduces a Graph Convolutional Network (GCN) to impose local topological structure constraints on the node representations. GCN performs graph-structure-based message propagation through a symmetrically normalized adjacency matrix, allowing nodes to integrate both their own features and those of their neighbors during the update process. Its propagation process can be formulated as:

(15) $$H^{\left(2\right)}=\sigma \left({\tilde{D}}^{-\frac{1}{2}}\tilde{A}{\tilde{D}}^{-\frac{1}{2}}H^{\left(1\right)}W_{g}\right)$$

where $H^{\left(1\right)}$ denotes the node representation matrix output by GraphSAGE; $\tilde{A}=A+I$ is the adjacency matrix with added self-loops (where A is the original adjacency matrix and I is the identity matrix); $\tilde{D}$ is the diagonal degree matrix of $\tilde{A}$, defined as ${\tilde{D}}_{ii}=\sum_{j}{\tilde{A}}_{ij}$; $W_{g}$ is the learnable weight matrix of the GCN layer; and σ(⋅) denotes the ReLU activation function. This symmetric normalization alleviates the influence of node degree variations and promotes more stable and comparable node representations within local neighborhoods.

In this right channel architecture, GraphSAGE and GCN form complementary local feature learning mechanisms: GraphSAGE, through explicit neighborhood sampling and aggregation operations, focuses on characterizing the expression synergies of gene nodes within their local interaction environments, making it suitable for capturing functional heterogeneity within local regulatory modules. In contrast, GCN introduces topological constraints through a graph-structure-based smoothing propagation process, ensuring that connected gene nodes maintain structural consistency in the representation space, thereby enhancing the stability and robustness of the embedding representations.

Finally, the node embedding representation output by the right channel is denoted as:

(16) $$z^{local}=h^{\left(2\right)}$$

This representation is used to characterize the local topological structure features of genes within the comprehensive gene network. Subsequently, this local structure embedding is concatenated with the global structure representation learned by the left channel along the feature dimension to jointly constitute the final gene representation. This provides multi-scale, complementary information support for the subsequent identification of key regulatory genes and the assessment of their regulatory potential.

### 2.4. Model Training Based on Self-Supervised Link Prediction and Gene Importance Assessment

After constructing a dual-channel graph neural network model, this paper uses a self-supervised learning strategy based on link prediction to train the model [42]. This allows the model to fully extract the potential structural information and functional relationships between genes in the gene network. This training framework uses the topology of the gene interaction network as an intrinsic supervision signal, and guides the model to learn biologically meaningful node embeddings by predicting the real connections between gene pairs.

#### 2.4.1. Definition of the Self-Supervised Link Prediction Task

After encoding via the dual-channel fused graph neural network, the final low-dimensional embedding representation of node $v_{i}$ is denoted as: $z_{i}\in R^{d}$ In the link prediction task, the model is required to determine whether a true interaction relationship exists between any pair of gene nodes $\left(v_{i},v_{j}\right)$. To this end, this study adopts the inner product function of node embedding vectors as the scoring function for predicting edge existence: $s_{ij}=z_{i}^{⊤}z_{j}$ where $(\cdot {)}^{⊤}$ denotes the vector transpose operation. A larger inner product value indicates higher similarity between the two genes in the embedding space, implying a stronger potential functional association.

#### 2.4.2. Strategy for Constructing Training, Validation, and Test Samples

During the model training process, the set of known gene interaction edges E is treated as the set of positive samples. To prevent the model from accessing validation or test information during training, this study employs a manual edge-level partitioning strategy, randomly dividing the original edge set into training, validation, and test sets with ratios of 90%, 5%, and 5%, respectively. During the model’s forward propagation, only the positive edges from the training set are used to construct the graph structure, ensuring that no information leakage occurs during the parameter update process. Positive edges in the validation and test sets are used exclusively for performance evaluation. Negative samples are generated online via a negative sampling mechanism. Specifically, in each round of training or evaluation, an equal number of negative edges are randomly sampled from all node pairs that do not have true connections, serving to construct binary classification supervisory signals. This strategy effectively reduces computational complexity while maintaining sample balance.

#### 2.4.3. Loss Function and Model Optimization Objective

Based on the aforementioned link prediction setting, the model training objective is formalized as a binary classification problem. For any node pair $\left(v_{i},v_{j}\right)$, the true label is defined as:

(17) $$y_{ij}=\{\begin{array}{ll} 1, & \mathrm{if}\;\left(v_{i},v_{j}\right)\in E^{+}\\ 0, & \mathrm{if}\;\left(v_{i},v_{j}\right)\in E^{-} \end{array}$$

This study employs the Binary Cross-Entropy with Logits Loss as the optimization objective for the model. The overall loss function is formulated as:

(18) $$L=-\frac{1}{\left|E^{+}\right|+\left|E^{-}\right|}\sum_{i,j}\left[y_{ij}\log\left(\sigma \left(s_{ij}\right)\right)+\left(1-y_{ij}\right)\log\left(1-\sigma \left(s_{ij}\right)\right)\right]\;$$

where σ(⋅) denotes the Sigmoid activation function, used to map the inner product scores into probabilities of edge existence. Model parameters are updated using the Adam optimization algorithm. During training, negative samples are dynamically generated in each iteration to enhance the model’s robustness and generalization capability.

#### 2.4.4. Model Evaluation Metrics

To comprehensively evaluate the model’s performance on the link prediction task, this study calculates several common binary classification metrics on the validation and test sets, including Accuracy, Precision, Recall, F1-score, Area Under the Receiver Operating Characteristic Curve (AUC), and Average Precision (AP). Among these, the AP metric demonstrates greater robustness to imbalanced positive and negative samples and is therefore adopted as the primary reference indicator for model selection and performance comparison.

#### 2.4.5. Gene Importance Scoring and Ranking Method

Upon completion of model training, the dual-channel fused graph neural network generates stable embedding representations for each gene node. Based on this embedding space, this study introduces an unsupervised gene ranking strategy to prioritize candidate genes within the comprehensive gene network.

Specifically, a representation-derived score is assigned to each gene node $v_{i}$, defined as the $L_{2}$ norm of its embedding vector: $I\left(v_{i}\right)=∥z_{i}{∥}_{2}\;$ where $∥\cdot {∥}_{2}$ denotes the Euclidean norm. This score reflects a mathematical property of the learned embedding space and characterizes the relative prominence of nodes in the representation space. It should be noted that this score does not directly correspond to biological regulatory importance, but rather serves as a heuristic measure for gene prioritization.

By ranking genes in descending order of this score, a set of candidate genes is obtained for downstream functional enrichment analysis and biological interpretation.

## 3. Results

### 3.1. Experimental Setup and Data Sources

This study utilizes the transcriptomic dataset GSE174508 from the GEO database, which covers maize under *Fusarium graminearum* infection conditions, as the primary data source. Initially, 1194 differentially expressed genes (DEGs) were screened based on differential expression analysis. Based on these DEGs, a Weighted Gene Co-expression Network Analysis (WGCNA) was constructed, yielding 281,891 co-expression edges to characterize the synergistic expression relationships between genes under infection conditions. Simultaneously, leveraging the standardized gene expression matrix, prior interaction gene networks were constructed by incorporating known protein-protein interaction information from the STRING database.

Building upon this, node-space alignment and edge-level fusion were performed on the WGCNA co-expression network and the STRING interaction network to obtain the final comprehensive gene network. The fused network comprises 22,861 gene nodes and 336,269 edges, integrating both condition-specific expression-driven information and stable biological interaction priors.

Model training employed a self-supervised link prediction task. Edges within the comprehensive gene network were randomly partitioned into training, validation, and test sets with a ratio of 0.9/0.05/0.05. During the training process, only positive edges from the training set were used to construct the graph structure. Negative samples were generated using a uniform random sampling strategy from unconnected node pairs in the network. Specifically, for each positive edge, one negative edge was sampled to maintain a balanced class distribution, which stabilizes optimization in the binary classification setting and avoids bias toward the majority class.

We note that this simple negative sampling strategy may introduce potential false negatives, as some randomly sampled node pairs could correspond to yet-undiscovered biological interactions. However, given the sparsity of gene interaction networks, the probability of sampling such true but unobserved edges is relatively low. Moreover, uniform negative sampling is widely adopted in graph representation learning due to its computational efficiency and scalability for large biological networks.

To further mitigate potential bias and improve sample diversity, negative edges were dynamically resampled during training across different epochs, reducing the risk of overfitting to a fixed set of negative patterns. Nevertheless, we acknowledge that more sophisticated strategies, such as structure-aware or hard negative sampling, may further improve model discrimination ability and will be explored in future work.

Regarding model parameter settings, the Adam optimizer was used for training with a learning rate of 0.01, a weight decay coefficient of 5 × 10^−4^, and a maximum of 200 epochs. These hyperparameters were determined based on a combination of preliminary experiments and commonly adopted settings in prior graph neural network studies. In particular, key parameters such as the learning rate and weight decay were selected from a reasonable range to ensure stable convergence and avoid overfitting.

A validation set (5% of the data) was used to monitor model performance during training, and the final parameter configuration was chosen based on validation results. This strategy ensures a balance between computational efficiency and model performance, while providing a fair basis for comparison across different models.

### 3.2. Link Prediction Performance Evaluation Results

To evaluate the representation learning ability of the proposed dual-channel fusion graph neural network in a comprehensive gene interaction network, we conducted link prediction experiments. The model first learns node embedding representations from the training set, and then predicts the potential interaction relationships between unknown gene pairs on the test set. We used six evaluation metrics—accuracy (Acc), precision (Prec), recall (Rec), F1 score, area under the curve (AUC), and average precision (AP)—to evaluate the model from both classification and ranking perspectives.

To ensure comprehensiveness and fairness in the comparison, we included several representative graph neural network models as baseline methods, including GCN, GraphSAGE, GAT, GATv2, and Graph Transformer.

The experimental results are summarized in Table 1. Overall, the proposed DC-FusionGNN exhibits stable and balanced performance on most evaluation metrics. Specifically, the model achieved an accuracy of 0.92, precision of 0.90, recall of 0.93, and an F1 score of 0.92, demonstrating strong classification capabilities in identifying potential gene interactions.

Different models exhibited varying trade-offs between precision and recall. For example, GCN and GATv2 had relatively high recall (0.98 and 0.95, respectively), but lower precision (0.64 and 0.79, respectively), indicating a tendency to produce more false positives. In contrast, the proposed model maintained a better balance between precision and recall, achieving the highest F1 score among all compared methods.

Among the baseline models, Graph Transformer achieved relatively competitive performance with an accuracy of 0.88 and an F1 score of 0.89, while GAT and GraphSAGE performed at a moderate level on most metrics. However, the proposed model still achieved higher accuracy and F1 score, indicating stronger robustness in identifying gene interaction relationships.

In terms of ranking performance, the proposed model achieved the highest AUC (0.95) and AP (0.94) among all models. Although GATv2 and Graph Transformer also demonstrated competitive ranking performance (AUC up to 0.94, AP up to 0.93), their overall classification performance was still slightly lower than the proposed model.

Furthermore, ablation experiments further validated the effectiveness of the dual-channel architecture. Using only the left channel, the accuracy, precision, recall, and F1 score were 0.88, 0.96, 0.79, and 0.87, respectively. Using only the right channel, these metrics were 0.88, 0.95, 0.80, and 0.87, respectively. In contrast, the dual-channel fusion model achieved further improvements in ranking metrics such as AUC and AP, demonstrating that fusing global dependency modeling capabilities with local structural information enables the learning of more discriminative node representations.

In summary, the proposed DC-FusionGNN exhibits competitive performance on most evaluation metrics compared to several baseline models. These results demonstrate that combining global dependency information with local structural features can more accurately and stably predict gene interaction relationships.

### 3.3. Model Performance Comparison and Effectiveness Analysis of the Dual-Channel Architecture

To evaluate the performance of the proposed dual-channel fusion graph neural network model in complex gene network prediction tasks, this study compared it with several representative graph representation learning methods, including GraphSAGE, GAT, GCN, GATv2, GraphTransformer, and their corresponding single-channel variants. All models were trained and evaluated under the same data partitioning and experimental settings. For comprehensive evaluation, multiple metrics were used, including accuracy (Acc), precision (Prec), recall (Rec), F1 score, area under the ROC curve (AUC), and average precision (AP).

Experimental results show that, at the optimal classification threshold of 0.60, the proposed model achieved an accuracy of 0.92, a precision of 0.90, a recall of 0.93, and an F1 score of 0.92. Furthermore, the model exhibits strong discriminative ability, with an AUC of 0.95 and an AP of 0.94, indicating its ability to effectively distinguish between real gene interactions and randomly generated negative edges.

**Figure 6:**
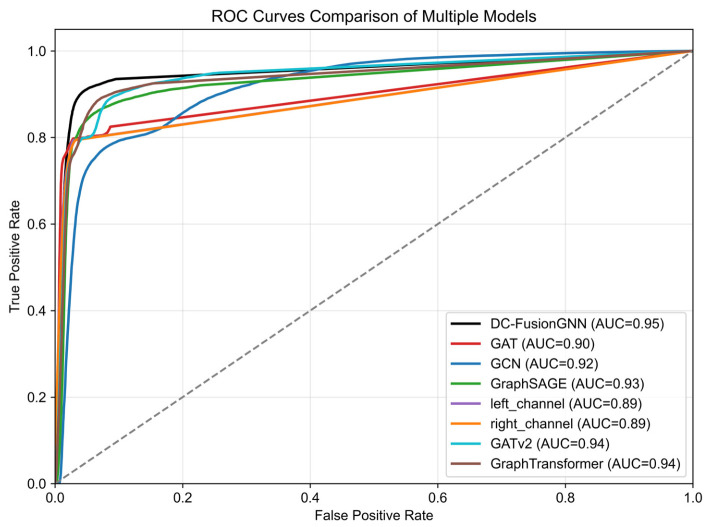
Comparison of ROC curves for different graph neural network models on the gene interaction prediction task. The proposed DC-FusionGNN achieved the highest AUC value among all compared methods, demonstrating superior discriminative ability and more effective representation learning performance.

Figure 6 shows a comparison of the ROC curves of different models. As shown in the figure, the proposed DC-FusionGNN outperforms baseline methods across most threshold ranges and achieves the highest AUC (0.95), surpassing GraphSAGE (0.93), GAT (0.90), GCN (0.92), the left-channel-only model (0.89), the right-channel-only model (0.89), GATv2 (0.94), and GraphTransformer (0.94). These results demonstrate that the dual-channel fusion strategy effectively integrates global structural dependencies and local topological information, thereby improving representation learning performance.

**Figure 7:**
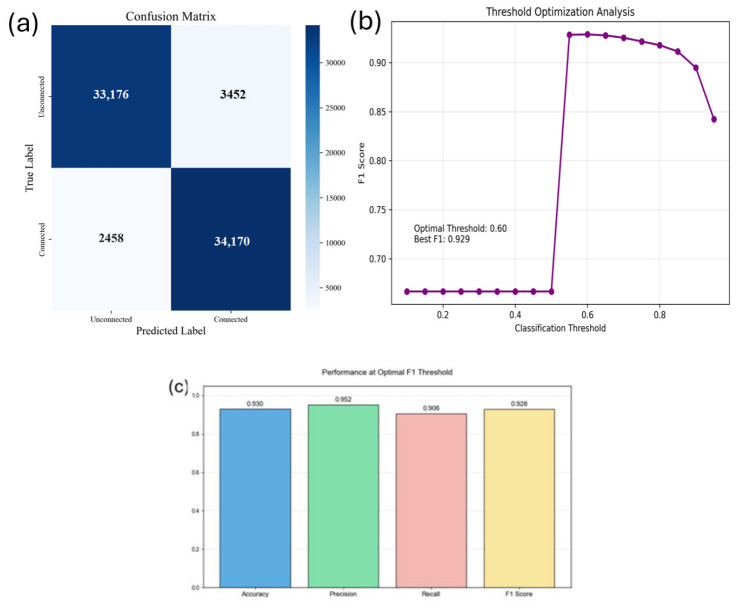
Performance evaluation of the DC-FusionGNN model on the test set. (**a**) Confusion matrix: Shows the classification results of positive and negative sample edges, correctly identifying 33,176 negative sample edges and 34,170 positive sample edges; (**b**) Threshold optimization analysis: The F1 score changes with the classification threshold, indicating that the model reaches the optimal balance point (optimal F1 ≈ 0.929) at a threshold of 0.60; (**c**) Performance metrics at the optimal threshold: At a classification threshold of 0.60, the model achieves an accuracy of 0.92, precision of 0.90, recall of 0.93, and an F1 score of 0.92.

Figure 7 further illustrates the detailed evaluation results of the proposed model. As shown in Figure 7a, the confusion matrix indicates that the model correctly identified 33,176 negative sample edges and 34,170 positive sample edges, exhibiting stable classification performance. The threshold optimization analysis in Figure 7b shows that the model achieves the best F1 score (approximately 0.929) when the classification threshold is set to 0.60, indicating a balance between precision and recall. In addition, Figure 7c summarizes the evaluation metrics under the optimal threshold setting, where accuracy, precision, recall and F1 score are all maintained at a high level.

**Figure 8:**
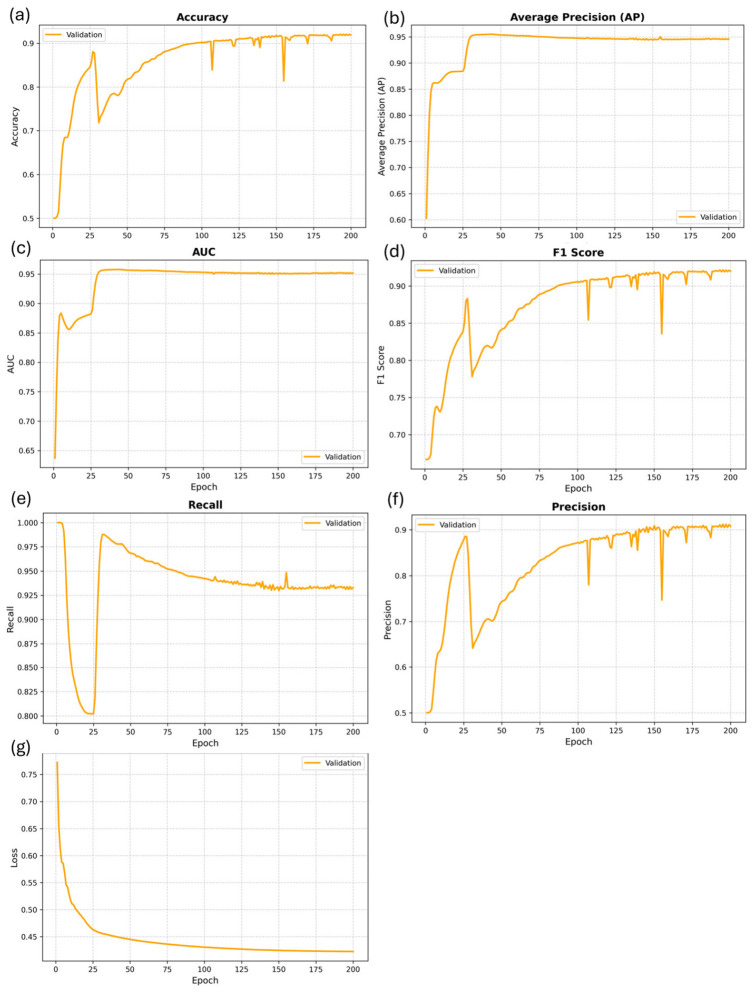
Variation trends of metrics during the model training process. The plots display the curves of Accuracy, Average Precision (AP), AUC, F1-score, Loss, Precision, and Recall on the validation set across training epochs. Results show that all performance metrics rise rapidly within the first 100 epochs and stabilize thereafter, with AUC and AP reaching high levels (0.95) early in the training. Concurrently, the Loss curve continuously decreases and converges to a low value. This indicates that the model possesses good convergence properties, effectively learning discriminative features from the gene network without significant overfitting.

Figure 8 illustrates the evolution of multiple evaluation metrics during the training process, where each metric is presented in an individual subplot (a–g) to improve clarity and readability. Specifically, Accuracy, Average Precision (AP), AUC, F1-score, Recall, Precision, and Loss are plotted as functions of training epochs.

As shown in Figure 8a–f, most performance metrics exhibit a rapid increase during the early training stage, followed by gradual stabilization after approximately 100 epochs, indicating effective convergence of the model. In particular, AUC and AP (Figure 8b,c) quickly rise to around 0.95 within the first 30–50 epochs and remain stable thereafter, demonstrating the model’s strong ability to capture discriminative structural patterns in the gene network.

The Loss curve in Figure 8g shows a consistent decreasing trend and gradually flattens as training progresses, further confirming the stability of the optimization process. Although minor fluctuations can be observed in certain metrics such as F1-score and Precision, the overall trends remain stable in later epochs.

Combined with the results presented in Figure 7, these findings indicate that the proposed dual-channel fused graph neural network effectively integrates global and local topological features, leading to robust node representations and consistently strong performance in gene interaction prediction tasks.

### 3.4. Robustness Experiment

To further evaluate the robustness of the proposed model against noise and unreliable interactions in the gene network, we conducted a perturbation analysis by randomly removing different proportions of edges (0%, 5%, 10%, 15%, 20%, 25%, and 30%) from the original network. The Area Under the Curve (AUC) was used as the primary evaluation metric.

**Figure 9:**
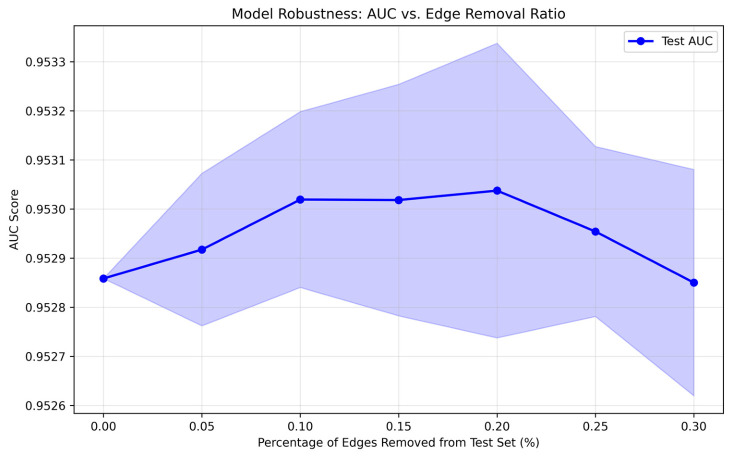
AUC performance of the model under varying edge removal ratios. Edges (0–30%) are randomly removed from the original network to simulate noise and incompleteness. The AUC remains consistently around 0.953 with only negligible variation (<0.001), indicating that the proposed model is highly robust to structural perturbations.

As illustrated in Figure 9, the model demonstrates remarkable stability under increasing levels of structural perturbation. Specifically, the AUC value remains consistently around 0.953 across all edge removal ratios. When the edge removal ratio increases from 0% to 20%, the model performance even shows a slight improvement, reaching a peak AUC of approximately 0.9530–0.9531. This phenomenon suggests that the model is not overly sensitive to certain redundant or potentially noisy edges, and may benefit from mild network sparsification.

As the perturbation level further increases to 25% and 30%, the AUC exhibits a minor decline. However, the decrease is marginal (less than 0.001 compared to the original network), indicating that the overall predictive performance is largely preserved even under substantial information loss. In addition, the relatively narrow fluctuation range across different perturbation levels further confirms the model’s stability.

These results demonstrate that the proposed model does not rely heavily on specific network connections derived from WGCNA and STRING, but instead captures robust and generalizable topological patterns within the gene network. Therefore, it exhibits strong tolerance to noise and missing edges, which is critical for real-world biological networks that are often incomplete and noisy.

In summary, the robustness experiment verifies that the proposed model maintains stable performance under varying degrees of network perturbation, highlighting its reliability and practical applicability in gene identification tasks.

Figure 8 shows trends in various metrics during model training. Figure 8a–f show the curves of accuracy (a), mean precision (b), AUC (c), F1 score (d), recall (e), precision (f), and loss (g) on the validation set as the number of training epochs changes. The results show that all performance metrics rise rapidly in the first 100 epochs and then stabilize, with AUC and AP reaching high levels (0.95) early in the training process. Meanwhile, the loss curve continues to decrease and converges to a low value. This indicates that the model has good convergence characteristics, can effectively learn discriminative features from the gene network, and does not exhibit significant overfitting.

### 3.5. Generalization Study on an Independent Dataset

To further evaluate the generalization ability of the proposed model, additional experiments were conducted on the independent dataset GSE39635. This dataset originates from *rice* (*Oryza sativa*), which differs from the original maize dataset in both species and biological background, providing a more rigorous test of cross-species transferability.

Without modifying the model architecture or hyperparameters, the trained model was directly applied to the GSE39635 dataset. Multiple evaluation metrics, including Accuracy, Precision, Recall, F1-score, Area Under the Curve (AUC), and Average Precision (AP), were used for comprehensive evaluation.

**Figure 10:**
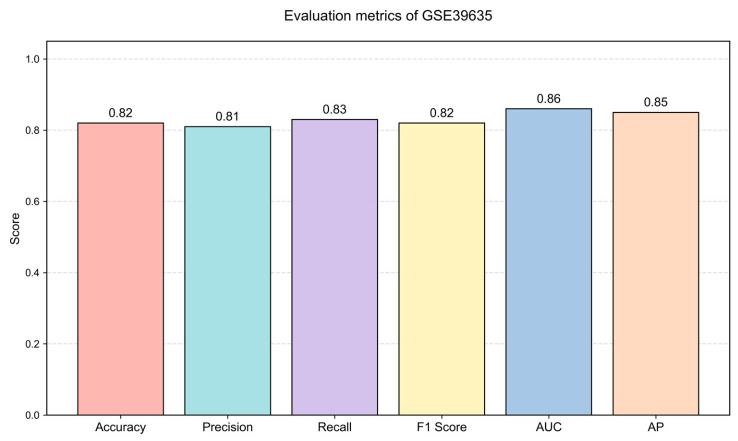
Performance of the proposed model on the GSE39635 rice dataset across multiple evaluation metrics. The model achieves balanced performance with Accuracy, Precision, Recall, and F1-score, along with strong discriminative ability as indicated by AUC and AP, demonstrating robust cross-dataset and cross-species generalization.

As shown in Figure 10, the proposed model achieves consistently strong and balanced performance across all evaluation metrics, with an Accuracy of 0.82, Precision of 0.81, Recall of 0.83, F1-score of 0.82, an AUC of 0.86, and an AP of 0.85.

**Figure 11:**
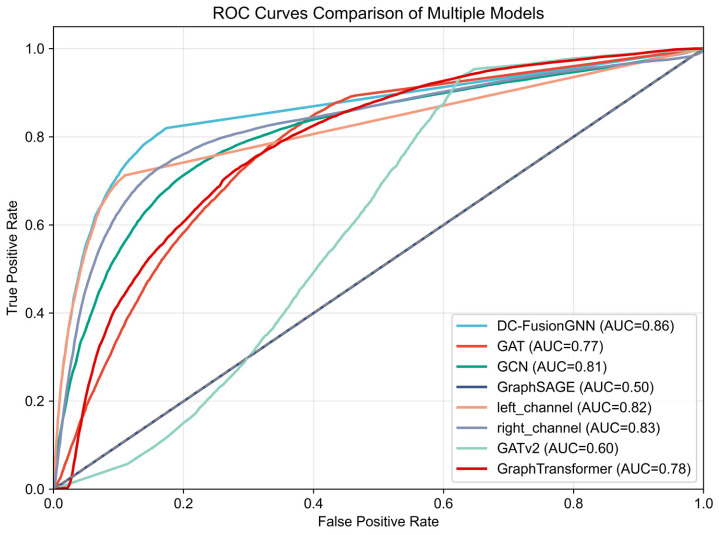
ROC curve comparison of DC-FusionGNN and the baseline model on the independent dataset GSE39635. The proposed DC-FusionGNN is directly evaluated on the cross-species dataset (Oryza sativa) without retraining or parameter tuning. The results show that DC-FusionGNN achieves the best overall discriminative performance, with the highest AUC (0.86), outperforming classic graph learning methods (GCN, GAT, GraphSAGE) and advanced variants (GATv2 and GraphTransformer). Furthermore, the two single-channel variants (left_channel and right_channel) also show good performance, but still lag behind the fusion model, highlighting the effectiveness of the dual-channel fusion strategy in achieving robust cross-dataset generalization.

To provide a more intuitive comparison of discriminative performance, a ROC curve comparison among all baseline methods and model variants is further presented in Figure 11. As illustrated, the proposed DC-FusionGNN consistently achieves the best performance, with the highest AUC value (0.86), outperforming GCN (0.81), GAT (0.77), GraphTransformer (0.78), and other variants such as GATv2 (0.60) and GraphSage (0.50). Notably, both single-channel variants (left_channel: 0.82, right_channel: 0.83) perform well, but remain inferior to the fused model, demonstrating the effectiveness of the dual-channel fusion strategy.

Importantly, despite significant differences between maize and rice in gene regulatory mechanisms and biological characteristics, the model maintains stable performance on the independent dataset. These results indicate that the proposed approach can capture robust and generalizable patterns in gene interaction networks.

Overall, the results demonstrate that the model is not limited to a specific dataset or species, but exhibits strong cross-dataset and cross-species generalization ability, further supporting its robustness and practical applicability.

### 3.6. Key Gene Ranking Results and Biological Significance Analysis

#### 3.6.1. Embedding-Based Gene Importance Analysis

To further investigate the biological relevance of the learned node representations, an embedding-based gene importance analysis was conducted on the candidate genes identified by the proposed dual-channel fusion graph neural network model. Specifically, the importance score of each gene was quantified using the L_2_ norm of its learned embedding vector, where genes with larger embedding norms were considered to exhibit stronger influence within the learned representation space.

**Figure 12:**
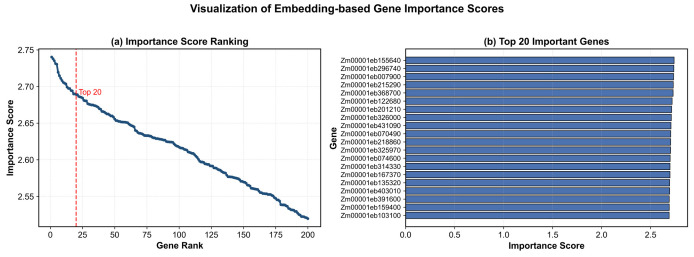
Visualization of embedding-based gene importance analysis. (**a**) Importance score ranking distribution of the top 200 candidate genes derived from node embedding norms. (**b**) Top 20 genes ranked according to embedding-based importance scores, where higher scores indicate stronger influence within the learned representation space.

Figure 12 presents the visualization results of the embedding-based importance scores. As shown in Figure 12a, the ranking distribution of the top 200 candidate genes exhibits a clear hierarchical pattern, where a relatively small subset of genes possesses substantially higher importance scores than the remaining candidates. This trend suggests that the proposed model effectively differentiates node influence during graph representation learning and topology reconstruction.

To further illustrate the ranking results, the top 20 genes with the highest importance scores are displayed in Figure 12b. These highly ranked genes are considered potential key regulatory candidates due to their stronger participation in structural information propagation and feature aggregation within the co-expression network.

It should be noted that the embedding norm does not directly represent biological regulatory strength; rather, it serves as a heuristic indicator reflecting the contribution of a node within the learned graph representation space. Nevertheless, the observed ranking distribution provides intuitive support for the interpretability and effectiveness of the proposed embedding-based importance scoring strategy.

Based on the ranked candidate genes, downstream GO and KEGG enrichment analyses were subsequently performed to further evaluate their potential biological significance.

#### 3.6.2. GO and KEGG Enrichment Analysis

**Figure 13:**
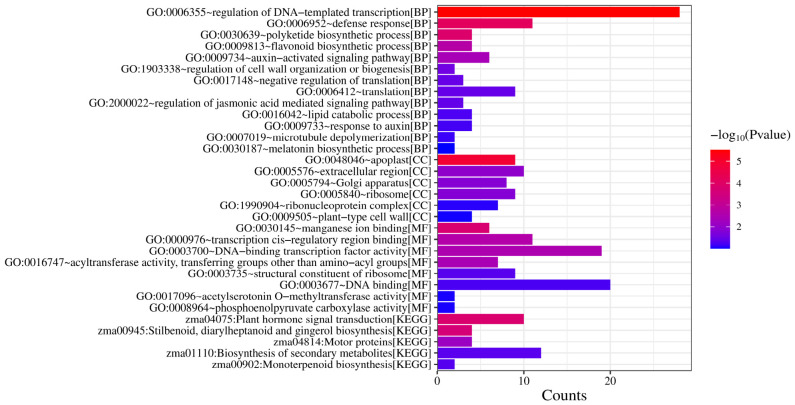
GO and KEGG enrichment analysis of the top 200 candidate genes. The enriched Gene Ontology (GO) terms, including biological process (BP), cellular component (CC), and molecular function (MF), as well as KEGG pathways are shown. The x-axis represents the number of genes (Counts) enriched in each term, and the color scale indicates the significance level (−log10 (*p*-value)).

Based on the node embedding representations learned by the dual-channel fused graph neural network model, this study ranked the genes within the comprehensive gene network by importance and screened out the top 200 candidate key genes. To further validate the biological relevance of the model’s screening results, Gene Ontology (GO) functional annotation and Kyoto Encyclopedia of Genes and Genomes (KEGG) pathway enrichment analyses were conducted on these candidate genes, as shown in Figure 13. The complete list of these 200 candidate genes has been made public in the GitHub (v1.0) repository.

The GO enrichment analysis results indicate that these candidate key genes are significantly enriched in multiple biological processes (BP) related to plant defense and regulation. Notably, terms such as “regulation of DNA-templated transcription” and “defense response” [43,44] are significantly enriched, suggesting that these genes may participate in plant immune responses by regulating transcriptional levels. Furthermore, several biological processes closely associated with plant metabolism and signal regulation, such as “flavonoid biosynthetic process,” “polyketide biosynthetic process,” “auxin-activated signaling pathway,” and “regulation of jasmonic acid mediated signaling pathway,” [45] also exhibit significant enrichment. This implies that these key genes may be involved in plant hormone signal regulation and secondary metabolic processes, thereby playing crucial roles in the response to pathogen infection [46].

At the cellular component (CC) level, these genes are primarily enriched in structural regions such as “apoplast” (*p* = 1.25 × 10^−5^), “extracellular region,” and “Golgi apparatus.” These cellular structures are closely linked to extracellular signal perception, substance transport, and defense responses in plants [47,48]. Meanwhile, regarding molecular function (MF), the enriched terms mainly include “DNA-binding transcription factor activity,” “transcription cis-regulatory region binding,” and “manganese ion binding” [49,50], indicating that these candidate genes play important roles in transcriptional regulation and protein function modulation.

KEGG pathway enrichment analysis further revealed that these key genes are significantly enriched in several signaling pathways related to plant stress resistance and metabolic regulation. “Plant hormone signal transduction” (zma04075, *p* = 1.01 × 10^−4^) is one of the most significant pathways, indicating that the plant hormone signaling network plays a pivotal regulatory role in the response to *Fusarium graminearum* infection [45]. Additionally, pathways related to plant secondary metabolism, such as “Stilbenoid, diarylheptanoid and gingerol biosynthesis” and “Monoterpenoid biosynthesis,” also show a certain degree of enrichment; these metabolites are typically closely associated with plant defense responses and disease resistance. Concurrently, the enrichment of the “Biosynthesis of secondary metabolites” pathway further suggests that secondary metabolic regulation may play a vital role in maize’s defense against pathogen invasion.

In summary, the results from GO functional annotation and KEGG pathway enrichment analyses demonstrate that the key genes screened by the dual-channel fused graph neural network model are primarily involved in biological processes closely related to plant immunity and stress response, including transcriptional regulation, plant hormone signal transduction, and secondary metabolism. These findings support the reliability of the model’s screening results from a biological perspective, indicating that the proposed method can effectively mine important genes with potential regulatory roles within the comprehensive gene network. This provides critical clues for further elucidating the molecular mechanisms underlying maize’s response to *Fusarium graminearum* infection.

#### 3.6.3. Functional Classification Statistics of Candidate Genes

**Figure 14:**
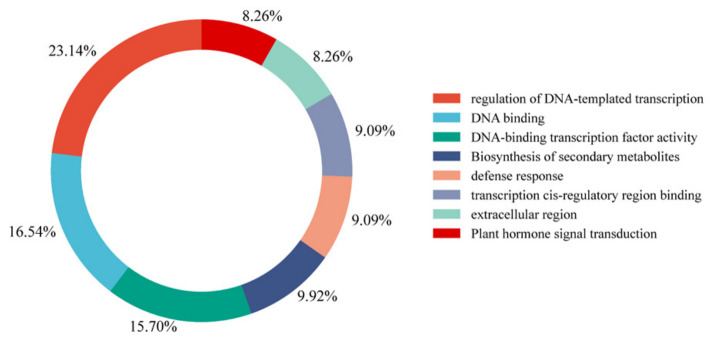
**Functional classification statistics of the top 200 screened maize candidate key genes.** The donut chart illustrates the distribution proportions of candidate genes across seven core functional modules. Results indicate that these genes are primarily concentrated in DNA Binding and Transcriptional Regulation (23.14%), Extracellular Region Localization (16.53%), and Secondary Metabolite Biosynthesis (15.70%). This distribution reflects the typical molecular defense strategy of maize in response to *Fusarium graminearum* infection, characterized by a highly synergistic network ranging from signal perception to transcriptional reprogramming and defense metabolic responses.

Based on the aforementioned enrichment analysis results, this study further conducted functional classification analysis on the top 200 maize candidate genes associated with *Fusarium graminearum* infection screened by the proposed model (Figure 14). The results indicate that these genes exhibit a coordinated defense-related functional architecture involving transcriptional regulation, signal perception, defense metabolism, and hormone-mediated immune responses, reflecting the typical molecular defense strategy of maize during *Fusarium graminearum* infection. Overall, these genes are primarily concentrated in the following functional categories:

- **Regulation of DNA-Templated Transcription (23.14%)**

This category represents the largest proportion among all candidate genes. Genes in this category are mainly involved in transcriptional regulation processes activated after pathogen infection. These genes may regulate the expression of downstream defense-related genes and participate in large-scale transcriptional reprogramming during the plant immune response.

- **DNA Binding (16.53%)**

Genes in this category mainly encode DNA-binding proteins involved in gene expression regulation and signal-responsive transcriptional control. These proteins may function as transcriptional regulators or cofactors that coordinate downstream defense signaling pathways.

- **DNA-Binding Transcription Factor Activity (15.70%)**

This category mainly includes genes encoding transcription factors such as members of the MYB, bHLH, NAC, and WRKY families. These transcription factors play essential regulatory roles in plant immunity by modulating the expression of defense-associated genes following pathogen invasion.

- **Biosynthesis of Secondary Metabolites (9.92%)**

Genes in this category are mainly associated with the biosynthesis of defense-related secondary metabolites, including flavonoids, phenolics, and terpenoid compounds. These metabolites often possess antimicrobial activities and contribute to the chemical defense system against fungal infection.

- **Defense Response (9.09%)**

Genes in this category directly participate in plant defense processes, including reactive oxygen species (ROS) metabolism, antioxidant regulation, and pathogen-responsive protein expression. These genes may play important roles in restricting pathogen invasion and enhancing host resistance.

- **Transcription Cis-Regulatory Region Binding (9.09%)**

This category includes genes involved in recognizing and binding transcriptional regulatory regions. These genes may participate in complex transcriptional regulatory networks that coordinate immune-related gene expression during pathogen infection.

- **Extracellular Region (8.26%)**

Proteins encoded by genes in this category are mainly localized in the extracellular space or cell wall-associated regions. These proteins may participate in pathogen recognition, extracellular signal perception, and early immune activation processes.

- **Plant Hormone Signal Transduction (8.26%)**

Some candidate genes are involved in plant hormone-mediated signaling pathways, which may contribute to balancing plant growth and immune defense during *Fusarium graminearum* infection. Hormone-regulated signaling networks are known to play important roles in coordinating stress responses in plants.

Overall, the 200 candidate genes screened by the proposed model were not randomly distributed but instead formed a highly coordinated defense-associated functional network. Extracellular proteins may function in pathogen signal perception, transcription factors may serve as central regulatory hubs, and downstream defense-related metabolic pathways may execute antimicrobial responses. These findings further support the effectiveness of the proposed model in identifying biologically meaningful disease resistance-related genes associated with maize resistance to *Fusarium graminearum* infection.

## 4. Discussion

This study addresses the identification of key regulatory genes in maize under *Fusarium graminearum* infection by proposing a dual-channel fused graph neural network model based on a comprehensive gene network. Through systematic comparative experiments and biological analyses, the results demonstrate that the proposed model exhibits significant advantages in both link prediction performance and key gene ranking tasks. This section further discusses the experimental results from the perspectives of model architecture design, feature learning mechanisms, and biological significance.

### 4.1. Effectiveness Analysis of the Dual-Channel Fused Modeling Strategy

Experimental results show that the proposed dual-channel fused model outperforms traditional single-channel graph neural network models, such as GCN and GraphSAGE, across multiple evaluation metrics including Accuracy, F1-score, AUC, and Average Precision. This indicates that in complex systems like comprehensive gene networks—characterized by high connectivity, high noise, and significant structural heterogeneity—relying solely on a single local neighborhood aggregation mechanism is insufficient to fully capture the multi-scale and multi-level regulatory relationships between genes.

Traditional graph convolutional models primarily achieve information propagation through feature smoothing over local neighborhoods, operating on the fundamental assumption that topologically adjacent nodes share high functional similarity. However, in the context of pathogen infection, maize transcriptional regulation often manifests as a systemic response spanning across modules and pathways. Relying exclusively on local topological information may lead to the over-smoothing or obscuring of critical signals. In contrast, by introducing a dual-channel structure, our model explicitly enhances the modeling capability for global expression dependencies while retaining the ability to model local structures, thereby significantly improving overall prediction performance.

### 4.2. Complementary Mechanisms of Global and Local Features

From an architectural perspective, the core advantage of the dual-channel design lies in the complementarity between global feature learning and local structural modeling. The left channel, based on structure-aware propagation and a global self-attention mechanism, is capable of modeling long-range dependency relationships between genes across the entire comprehensive gene network. This facilitates the capture of potential synergistic regulatory patterns between different functional modules. Such global modeling capability is particularly crucial for characterizing the widespread phenomenon of systemic transcriptional reprogramming observed during *F. graminearum* infection.

In comparison, the right channel employs local topological structure learning strategies based on GraphSAGE and GCN, focusing more on depicting the structural position of genes within local interaction networks and their neighborhood expression synergies. GraphSAGE preserves node individuality through explicit neighborhood feature aggregation, while GCN’s normalized message propagation mechanism further introduces local topological constraints, ensuring that genes with similar functions or topological structures possess more consistent representations in the embedding space.

Experimental results indicate that model performance declines when using either channel alone. However, optimal performance in both link prediction and key gene identification tasks is achieved when fusing these two types of representations at the feature level. This phenomenon further validates the complementary value of global expression dependency information and local topological structural information in modeling comprehensive gene networks.

### 4.3. Biological Rationality of Key Gene Ranking Results

Based on the gene embedding representations learned by the dual-channel model, this study further quantified the importance of gene nodes and screened the top 200 candidate key genes. GO and KEGG functional enrichment analyses of these genes revealed significant enrichment in numerous biological processes and pathways related to plant defense responses, signal transduction, and stress responses. These findings indicate that the model’s screening results possess strong biological consistency.

This result suggests that the proposed method not only improves network representation learning at the computational level but also effectively mines core genes that may play pivotal regulatory roles during pathogen infection from the comprehensive gene network. These genes hold promise as important candidates for subsequent functional validation experiments and disease resistance mechanism studies, providing new clues for elucidating the systemic response mechanisms of maize to *F. graminearum* infection.

### 4.4. Method Advantages, Limitations, and Future Work

Overall, the proposed dual-channel fusion graph neural network model demon-strates good performance and application potential in constructing comprehensive gene networks and identifying key regulatory genes. However, the model still has some limitations. First, the model is somewhat dependent on the quality of the input network structure; if the prior interaction network or co-expression network contains a large number of noisy edges, it may affect the stability of the embedding representa-tion. Second, the current gene importance scoring is mainly based on static network embeddings and does not explicitly consider the dynamic changes in regulatory rela-tionships at different infection time points or under different conditions. Third, regarding the negative sampling protocol, the current implementation employs uniform random sampling with epoch-wise resampling. While this is widely adopted and ensures fair model comparison, recent studies have shown that structure-aware [51] and hard negative mining [52] strategies can further improve discrimination ability in graph representation learning. A systematic comparison of these strategies on biological networks—where the distribution of “true negatives” is itself biologically meaningful and not yet fully characterized—represents an important direction for our follow-up work. In addition, although we have validated the contribution of the dual-channel architecture at the channel level, more fine-grained component-level ablation studies—such as removing the Trans-former self-attention in the left channel, disabling LayerNorm, and decomposing the right channel into GraphSAGE-only and GCN-only variants—have not yet been con-ducted and will be explored in future work.

In future work, we plan to integrate time-series transcriptome data to construct a dynamic graph neural network model, aiming to characterize the dynamic reconstruction process of regulatory networks during pathogen infection. Furthermore, integrating experimental validation data or causal inference methods is expected to further improve the biological credibility and interpretability of key gene identification results. In addition, we will explore an adaptive fusion strategy based on attention mechanisms to dynamically assign weights to global and local features, which may further improve the model’s flexibility and performance.

## 5. Conclusions

In this study, we developed a dual-channel fusion graph neural network framework to address the challenges of modeling complex and multi-scale gene regulatory interactions in maize under *Fusarium graminearum* infection. By combining a co-expression network based on weighted gene co-expression network analysis (WGCNA) with a gene network mapped from a protein-protein interaction network based on STRING, a comprehensive gene network was constructed, providing a biologically meaningful structural basis for it.

The proposed framework employs a dual-channel architecture to capture complementary information from the gene network. The global channel combines structure-aware propagation with Transformer-based self-attention to model long-range and cross-module dependencies among genes, while the local channel integrates GraphSAGE and graph convolutional networks to characterize neighborhood topology and local regulatory patterns. The fusion of global and local embeddings enables the model to achieve multi-scale representation learning of gene regulatory features.

Through a self-supervised link prediction strategy, the model learns informative and robust node embeddings by leveraging supervision signals automatically derived from the graph structure (i.e., observed edges and sampled non-edges). Experimental results show that the proposed method achieves competitive and overall improved performance compared with several baseline graph neural network models across multiple evaluation metrics, including Accuracy, F1-score, AUC, and Average Precision.

Further analysis of the top-ranked genes revealed that they were significantly enriched in biological processes and pathways related to plant defense responses, including transcriptional regulation, hormone signaling, and secondary metabolism. These findings support the potential biological validity of the candidate genes and demonstrate that the proposed framework can capture relevant regulatory signals.

In summary, this study provides a multi-scale and structure-aware computational framework for key gene identification in complex gene networks. The proposed framework offers new insights into the molecular mechanisms underlying maize responses to *Fusarium graminearum* infection and demonstrates stable performance across datasets, suggesting its potential for cross-species application. It may therefore serve as a useful framework for gene discovery in other biological systems.

Future work will focus on integrating multi-omics data and incorporating experimental validation to further elucidate the functional roles of the identified genes and improve the general applicability of the model.

## Figures and Tables

**Table 1:** Performance comparison of different graph neural network models on the GSE174508 dataset. All results are averaged over 5 independent runs. The bold values indicate the best performance, and underlined values denote the second-best results in each column. The evaluation metrics include Accuracy, Precision, Recall, F1-Score, Area Under the ROC Curve (AUC), and Average Precision (AP). Baseline methods include GCN, GraphSAGE, GAT, GATv2, and Graph Transformer. Left-Channel and Right-Channel represent variants utilizing single-channel features, while Dual-Channel denotes the proposed multi-channel fusion approach.

Algorithm Name	Accuracy	Precision	Recall	F1-Score	AUC	AP (Average Precision)
GraphSAGE	0.84	0.79	0.92	0.85	0.93	0.91
GAT	0.87	0.91	0.82	0.86	0.90	0.89
GCN	0.72	0.64	0.98	0.78	0.92	0.88
GATv2	0.85	0.79	0.95	0.86	0.94	0.93
GraphTransformer	0.88	0.85	0.92	0.89	0.94	0.93
Left-Channel	0.88	0.96	0.79	0.87	0.89	0.88
Right-Channel	0.88	0.95	0.80	0.87	0.89	0.88
DC-FusionGNN	0.92	0.90	0.93	0.92	0.95	0.94

## Data Availability

The datasets analyzed in this study are publicly available in the NCBI Gene Expression Omnibus (GEO) database under accession number GSE174508. The code used for model implementation and analysis is available at: https://github.com/qmj20/DC-FusionGNN/tree/master (accessed on 24 March 2026).

## Abbreviations

The following abbreviations are used in this manuscript:

AP	Average Precision
AUC	Area Under the Curve
BP	Biological Process
CC	Cellular Component
DEGs	Differentially Expressed Genes
GCN	Graph Convolutional Network
GEO	Gene Expression Omnibus
GNN	Graph Neural Network
GO	Gene Ontology
GSR	Gibberella Stalk Rot
KEGG	Kyoto Encyclopedia of Genes and Genomes
MF	Molecular Function
PPI	Protein–Protein Interaction
PR	Pathogenesis-Related
RLK	Receptor-Like Kinase
RNA-seq	RNA sequencing
ROS	Reactive Oxygen Species
STRING	Search Tool for the Retrieval of Interacting Genes/Proteins
TOM	Topological Overlap Matrix
WGCNA	Weighted Gene Co-expression Network Analysis
GraphSAGE	Graph Sample and Aggregate
